# Risk factors associated with the practice of child marriage among Roma girls in Serbia

**DOI:** 10.1186/s12914-016-0081-3

**Published:** 2016-02-01

**Authors:** David R. Hotchkiss, Deepali Godha, Anastasia J. Gage, Claudia Cappa

**Affiliations:** Department of Global Community Health and Behavioral Sciences, Tulane University, New Orleans, LA USA; Independent Consultant, Indore, India; UNICEF, New York, NY USA

## Abstract

**Background:**

Relatively little research on the issue of child marriage has been conducted in European countries where the overall prevalence of child marriage is relatively low, but relatively high among marginalized ethnic sub-groups. The purpose of this study is to assess the risk factors associated with the practice of child marriage among females living in Roma settlements in Serbia and among the general population and to explore the inter-relationship between child marriage and school enrollment decisions.

**Methods:**

The study is based on data from a nationally representative household survey in Serbia conducted in 2010 – and a separate survey of households living in Roma settlements in the same year. For each survey, we estimated a bivariate probit model of risk factors associated with being currently married and currently enrolled in school based on girls 15 to 17 years of age in the nationally representative and Roma settlements samples.

**Results:**

The practice of child marriage among the Roma was found to be most common among girls who lived in poorer households, who had less education, and who lived in rural locations. The results of the bivariate probit analysis suggest that, among girls in the general population, decisions about child marriage school attendance are inter-dependent in that common unobserved factors were found to influence both decisions. However, among girls living in Roma settlements, there is only weak evidence of simultaneous decision making.

**Conclusion:**

The study finds evidence of the interdependence between marriage and school enrollment decisions among the general population and, to a lesser extent, among the Roma. Further research is needed on child marriage among the Roma and other marginalized sub-groups in Europe, and should be based on panel data, combined with qualitative data, to assess the role of community-level factors and the characteristics of households where girls grow up on child marriage and education decisions.

**Electronic supplementary material:**

The online version of this article (doi:10.1186/s12914-016-0081-3) contains supplementary material, which is available to authorized users.

## Background

### Rationale

In many low- and middle-income countries, child marriage is extensive, with one-third of girls being married before age 18 and one in nine being married before age 15 [1]. A practice that is driven by poverty, social norms, and discrimination against girls, child marriage has emerged as an important social issue in recent years due in part to increased concerns among reproductive health advocates about the harmful consequences for young women marrying too early [2, 3]. These consequences can include: dropping out of school; health risks that result from early sexual activity and pregnancy, including sexually transmitted diseases and maternal mortality; being prevented from taking advantage of economic opportunities; and if they have children, child malnutrition and mortality. There is also concern that child marriage deprives girls of their basic human rights and puts them at risk for harmful practices and disadvantage, including exploitation, intimate partner violence, and abuse [4]. Given these concerns, there is increased interest in efforts to empower children and adolescent girls in low- and middle-income countries in order to protect their human rights and the overall wellbeing of women and children.

Most empirical research on the issue of child marriage has focused on South Asian and sub-Saharan countries that have a very high percentage of females who first marry before age 18 [1, 5]. Relatively little research has been conducted in Central and Eastern European countries where the prevalence of child marriage on average is relatively low among the overall population, but relatively high among marginalized ethnic sub-groups, particularly the Roma. A case in point is Serbia, a country in Central Europe with an estimated population of 7.2 million in 2013 and a Roma population which is estimated to range from 100 thousand to 500 thousand [6]. In 2010, about half (50.4 %) of Roma women 20 to 24 years of age reported first marrying before age 18, compared to 5.0 % of women of the same age group living in the general population in Serbia [7].

Although the issues of child marriage and the wellbeing of Roma girls have received increased attention in recent years, there is relatively little empirical evidence available regarding the determinants of child marriage among the Roma in European countries. Evidence on these issues can be useful to improve the design and targeting of adolescent health and human rights strategies.

### Purpose

The purpose of this study is to assess the risk factors associated with the practice of child marriage among females living in Roma settlements in Serbia, and to make comparisons with females living among the general population. The study is based on two population-based household surveys conducted in 2010 by the Statistical Office of the Republic of Serbia with support from the United Nations Children’s Fund (UNICEF) – a nationally representative survey of women of reproductive age and a survey representative of women living in Roma settlements. For each survey, we conducted a bivariate analysis of the association of child marriage with a number of indicators of socio-economic status, including education, household wealth, and religion, among women 20 to 24 years of age, and a multivariate analysis of the determinants of child marriage and current school enrollment status among girls 15 to 17 years of age. In addition, we investigated whether child marriage was associated with attitudes related to domestic violence. The research presented in this article is part of a larger research study funded by UNICEF that empirically assessed the child marriage situation in 12 countries in Europe, Africa, Asia, and Latin America and the Caribbean based on data from publically available Demographic Health Surveys and Multiple Indicator Cluster Surveys.

### Description of the Roma population

Approximately 5.2 million Roma are estimated to live in Central and Eastern Europe, with over 108,000 defining themselves as Roma in Serbia. The Roma in Serbia consist of three groups: Roma who have lived in the country their entire lives; internally displaced persons as a result of the crisis in Kosovo during the late 1990’s; and returnees from Western Europe [8].

Roma are widely considered to be a marginalized population in Serbia and the rest of the region in that they are subject to stereotyping and discrimination based on their ethnicity, their physical features, and their low socio-economic status [8]. On average, the Roma in Serbia have lower levels of education, higher rates of unemployment, and poorer health outcomes than the general population. In, 2010, only 13.9 % of Roma females 15 to 49 years of age had completed secondary school compared to 57.0 % of Serbian females [7]. According to the World Bank, as few as one in five Roma were employed in 2010 with an employment gap of 29 percentage points between Roma and non-Roma populations [9], and in 2007, Roma women were over three times more likely to self-report their health status as being poor compared to non-Roma women [10].

### Roma marriage practices

Roma women are expected to marry, have children, and care for their husbands and children. Although the legal age of marriage is 18 in Serbia [11], many females enter marriage at much younger ages. According to the Statistical Office of the Republic of Serbia, 50.5 % of Roma women 20 to 24 years of age in 2010 reported marring before age 18 (down from 54.1 % 2005–06). For women in the same age group in the general population, only 5.0 % reported first marrying below age 18 (down from 5.7 % in 2005–06) [7]. In addition to social norms, marriage decisions among the Roma can be influenced by economic incentives in the form of bride price – in which the groom’s family pays the bride’s family a price for a marriageable girl. Previous qualitative studies indicated that the bride price paid among the Roma was determined by multiple factors, including whether the bride was a virgin, the appearance of the bride, the reputation of the family, the wealth and property status of both the groom’s and bride’s family, and the level of acquaintance between the two families [12, 13]. According to Cvorovic (2004), the practice of paying bride price is still common within Roma settlements in Serbia [13].

## Methods

Based on a review of the analytical literature on child marriage [4, 14–20], we developed a conceptual framework to guide the analysis. The framework posits that country-, community-, household-, and individual-level factors can play crucial roles in determining whether households decide to marry off their daughters prior to the legal age of marriage. The framework also hypothesizes that household decisions regarding whether to marry daughters as children and school enrollment are made simultaneously, not sequentially, to allow for the possibility that “those who intend to marry later (for whatever reason) tend to stay in school longer and those who intend to marry early leave school earlier to do so” [3]. Our endogenous treatment of these two types of decisions follows Mensch et al. (2006) and Bajracharya & Amin (2010) [3, 14]. It also follows the spirit of Becker’s application of neo-classical household economic theory to the analysis of marriage [21].

Household- and individual-level determinants of child marriage include household wealth and income, the educational attainment of parents and relatives, religion, ethnicity, the age of the child and, and for girls, whether menarche has occurred. In addition to factors identified as important by economists, the framework incorporates factors emphasized by researchers from other disciplines, such as sociology (social norms, social networks) and anthropology (kinship systems). Community-level factors encompass social norms, which are typically defined as prevailing expectations within the community of appropriate behavior, or attitudes of approval and disapproval, specifying what ought and ought not to be done. These social norms include norms regarding gender, including child-rearing practices for boys and girls, and socialization. Also important is the concept of agency. Who in the household is making marriage decisions for children, and what role does the household have above and beyond the influence of community factors? Within a given community, it is assumed that households vary in terms of their willingness to marry their daughters early as a risk mitigation strategy – to broaden kinship in order to cope with the possibility of future health and economic shocks. The mother or other female relatives in the household may also have varying degrees of empowerment and attitudes regarding marriage, which can also be important. All of these factors can potentially influence not only the household decision of whether to marry off their daughters as a child, but also education decisions.

The primary source of data for the study is the 2010 Serbia Multiple Indicator Cluster Survey (MICS), which included a nationally representative sample of women of reproductive age throughout the country and a separate representative of women living in Roma settlements. The rationale for conducting a survey of Roma settlements to assess the wellbeing of Roma women and children, rather than oversampling persons who identify themselves as Roma in the national survey, is that many Roma may not define themselves as Roma due to fear of discrimination. In order to overcome this problem, a separate MICS of households living in Roma settlements was conducted, where it was assumed, based on previous research and government sources, that the vast majority of persons living in these settlements are in fact Roma [7]. For the general household survey, data were collected from 6,392 households and 5,797 women of reproductive age (15 to 49 years of age). For the Roma settlements survey, data were collected from 1,711 households and 2,118 women of reproductive age. Ethical approval was sought and granted by the Statistical Office of the Republic of Serbia, based on the Law on Personal Data Protection. Informed consent procedures were administered to all survey respondents. Further details about the methodology and data collection procedures can be found in Statistical Office of the Republic of Serbia (2011) [7]. The MICS data sets are publically available upon request by UNICEF.

Using the data from both surveys, both bivariate and multivariate analyses were carried out. The bivariate analyses were restricted to women 20–24 years of age and used to investigate the associations between child marriage (as measured by two dichotomous indicators – whether the individual reported entering a formal marriage or informal union before age 18 and before age 15) and the following household- and individual level characteristics: urban/rural residence, region, religion, household wealth, and educational attainment. We also investigated the bivariate associations between child marriage and attitudes towards domestic violence. The MICS included five questions on attitudes towards domestic violence. Questions were asked on whether the woman believes it is justifiable for a husband to beat his wife if she a) goes out without telling her husband, b) neglects the children, c) argues with her husband, d) refuses sex with her husband, and e) burns the food. Unfortunately, women were not asked whether they have ever been victims of domestic violence.

Multivariate analysis was conducted to explore how individual-, household-, and community-level factors were associated with child marriage and school enrollment among girls age 15 to 17 years. This age group was chosen because it is the only age group in which we had information on both marital status and school enrollment. The specific type of model we estimated was a bivariate probit model in which the outcome variables of interest were current marital status and school enrollment. Two separate models were estimated: one for girls from the nationally representative sample, and another for girls from the sample of households living in Roma settlements. For each model, the two dependent variables were marital status, as measured by a dichotomous variable of whether the girl was currently married or in union, and school enrollment, as measured by a dichotomous variable of whether the girl attended school in the current year.

There are two advantages of using a simultaneous modeling approach, as compared to a sequential modeling approach that estimates the probability of child marriage as a function of educational attainment. First, if there is interdependence among marriage and education decisions, modeling one outcome as a function of another may lead to biased parameter estimates of the association between education and the prevalence of child marriage. Second, the cross-sectional nature of the MICS does not permit the investigation of the sequential process of household decision-making regarding marriage and school enrollment decisions.

The independent variables consist of the age of the girl, relative household wealth (based on wealth quintiles generated through principle components analysis of household assets), religion, and urban/rural status. In addition, we included the following community-level measures in the models:

- Percent of sampled adult females in the community who have zero tolerance of wife beating. This was measured by the percent of women in the community who *do not* justify wife beating for all five reasons: if wife goes out without telling husband; if wife neglects the children; if wife argues with husband; if wife refuses to have sex with husband; if wife burns the food.
- Percent of sampled adult females in the community with secondary or higher levels of education.
- Percent of sampled adult females in the community who were first married before age 18.

The sampling cluster was used to define the community, and in generating the community-level measures, we excluded the reference woman from the calculations to ensure that her characteristics did not influence the values of the indicators.

Descriptive statistics for the variables included in the model are presented in Table 1.

**Table 1:** Descriptive statistics for variables included in Bivariate Probit models

	Roma	General population
Variable	%	%
Residence		
Urban	64.89	53.64
Rural	35.11	46.36
Religion		
Orthodox Christians	56.58	87.53
Others	43.42	12.47
Household wealth		
Poorest	19.06	14.84
Poorer	20.19	18.68
Middle class	19.31	27.84
Richer	22.07	20.19
Richest	19.37	18.44
Mean community-level indicators among adult females		
Secondary or higher levels of education	12.56	82.87
First married before age 18	62.77	9.66
Zero tolerance of wife-beating	75.41	96.43
N	221	254

%: Weighted percentage

In the next section, we first present the results on the prevalence of child marriage among females 20 to 24 years of age for both the general population and those living in Roma settlements. To explore whether the extensiveness of the practice has changed over time, we then report the prevalence among older birth cohorts of females. We then report the results on the spousal age gap among females 15 to 19 and 20 to 24 years of age and on attitudes towards domestic violence. Next, we show patterns of child marriage practice among Roma females by various indicators of socio-economic status. To conclude the section, we present the multivariate results of the determinants of child marriage among females 15 to 17 years of age for both the nationally representative sample and the Roma settlements sample.

## Results

### Prevalence

**Fig. 1:**
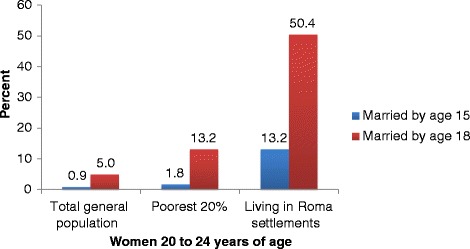
Percent of women ages 20 to 24 who report first marrying before ages 15 and 18, 2010

Roma girls in Serbia were found to be at very high risk of being married as children. In 2010, 50.4 % of women 20 to 24 years of age reported being first married before age 18 (Fig. 1) and 13.2 % were first married before age 15. This is considerably higher than the rates among females of the same age in the general population (5.0 % first married before age 18, 0.9 % first married before age 15), and among females living in the poorest 20 % of households in the general population (13.2 % and 1.8 % respectively). (See Additional file 1 for 95 % confidence intervals for estimates of the percentages first marrying before age 18).

### Spousal age gap

**Fig. 2:**
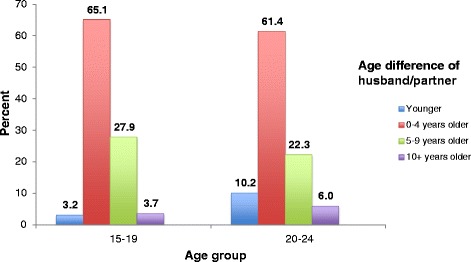
Percent distribution of currently married/in union females ages 15–19 and 20–24 years living in Roma settlements by age difference with her husband/partner, 2010

The difference in the ages of females who marry young and their partners can be an indication of the female’s vulnerability in the marriage [1]. The vast majority of currently married or in union Roma females aged 15–19 years and 20–24 years in 2010 had spouses less than five years older than themselves (Fig. 2). In 2010, 68.3 % of girls 15–19 years of age reported having a spouse less than five years older or a spouse that was younger, and 71.6 % of women 20–24 years of age reported the same. Overall, the spousal age gap among Roma females 15 to 24 years of age was much lower than that found among currently married females in the general population. For example, among currently married women 20 to 24 years of age in the population, 50.5 % had husbands who are five or more years older (results not shown), compared to 28.3 % among currently married Roma women in the same age group.

### Patterns by socio-economic status

**Fig. 3:**
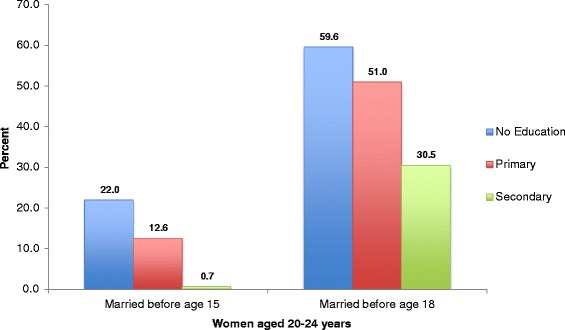
Percent of women living in Roma settlements age 20–24 first married before ages 15 and 18, by educational attainment, 2010

The prevalence of child marriage among the Roma varied by a number of socio-economic and geographic characteristics. With respect to education, 22.0 % of Roma females aged 20–24 years with no formal education and 12.6 % of those with only primary education were married at 14 years of age or younger (Fig. 3). Using the older age of first marriage threshold, the prevalence of marriage before age 18 decreases with increasing education, though remains high even among those with increased education attainment. In 2010, 59.6 % of females with no formal education and 51.0 % of those with only primary education were first married before age 18, compared to 30.5 % of those with secondary education. Prevalence estimates for child marriage among women attaining higher levels of education are not reported due to insufficient sample sizes (*n* < 25).

**Fig. 4:**
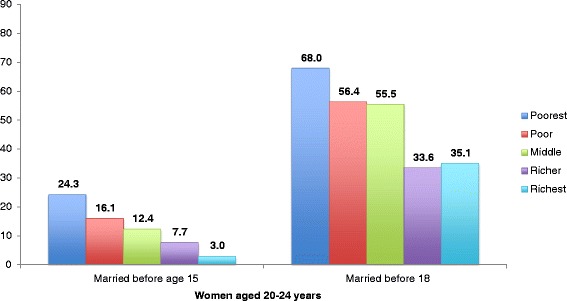
Percent of women living in Roma settlements ages 20–24 married before ages 15 and 18, by wealth group, 2010

The practice of child marriage also varied by relative household wealth. In 2010, 24.3 % of females living in the poorest quintile of households were married by age 15, compared to 12.4 % of those in the middle wealth group and 3.0 % of those in the richest wealth group (Fig. 4). Using the older age of first marriage threshold, 68.0 % of Roma women aged 20–24 years in the poorest wealth group were married before age 18, compared to 55.5 % in the middle wealth group and 35.1 % in the richest wealth group.

Among Roma women 20 to 24 years of age, the practice of child marriage was also found to be more prevalent in rural areas than in urban areas (63.8 % vs. 54.1 %, results not shown) and among non-Orthodox women than Orthodox women (54.5 % vs. 47.3 %, results not shown).

### Attitudes towards domestic violence

We found that a lower percentage of women first married before age 15 or age 18 had zero tolerance of wife beating (15.8 and 15.3 %, respectively) than women first married at age 18 or older (18.1 %), but the differences were not statistically significant.

### Bivariate probit results

Table 2 presents the results of the bivariate probit estimations of the determinants of being currently married and enrolled in school during the current year based on the nationally representative sample and the Roma settlements sample of 15 to 17 year old girls. Because this part of the analysis is based on females in a three-year age band, the number of observations used for the models was small (253 for the model based on girls in the general population and 198 for the model based on girls living in Roma settlements). For the model based on the general population, residence in communities with a higher prevalence of child marriage among adult women was positively associated with being currently married (*p* = 0.02). The other two community-level factors, the percentages of women with higher education and with zero tolerance for wife beating were not statistically significant at the *p* = 0.05 level (*p* = 0.08 and *p* = 0.09, respectively). With respect to the individual-level and household-level factors, age was found to be statistically significant and girls who lived in the second to poorest, and richest quintiles of households were significantly less likely to have been currently married than girls living in the poorest wealth quintile, after controlling for other factors.

**Table 2:** Bivariate Probit results of the determinants of current marital status and school enrollment status, all girls 15–17 years (general population sample)

	General population		Roma	
Variable	Coefficient	S.E.	Coefficient	S.E.
Currently married				
Rural (reference = urban)	0.15	0.41	0.58*	0.30
Religion (reference = Orthodox Christians)	0.45	0.46	0.23	0.27
Wealth (reference = poorest wealth quintile)				
Second wealth quintile	-1.10**	0.54	-0.21	0.41
Third wealth quintile	-1.11*	0.60	0.27	0.36
Fourth wealth quintile	0.02	0.55	-0.52	0.44
Richest wealth quintile	-9.18***	0.80	-0.69	0.43
Age (reference = 17 year olds)				
15 years of age	-2.65***	0.72	-1.52***	0.30
16 years of age	-2.16***	0.58	-0.24	0.30
Community % of women with secondary or higher education	-0.03*	0.01	-0.01	0.01
Community % of women married under age 18	0.05**	0.02	0.01	0.01
Community % of women with zero tolerance of wife beating	-0.04*	0.02	0.00	0.01
Constant	3.44	2.30	-0.97	1.09
Currently enrolled in school				
Rural (reference = urban)	0.34	0.32	-0.36	0.29
Religion (reference = Orthodox Christians)	0.20	0.44	-1.06***	0.31
Wealth (reference = poorest wealth quintile)				
Second wealth quintile	0.67	0.49	-0.16	0.50
Third wealth quintile	1.33***	0.48	0.62	0.48
Fourth wealth quintile	0.74	0.55	0.86*	0.49
Richest wealth quintile	8.61***	0.75	1.36**	0.52
Age (reference = 17 year olds)				
15 years of age	1.16***	0.33	0.89	0.31
16 years of age	0.22	0.46	0.26	0.32
Community % of women with secondary or higher education	0.02***	0.01	0.00	0.01
Community % of women married under age 18	-0.03	0.02	-0.01	0.01
Community % of women with zero tolerance of wife beating	0.02	0.02	-0.02	0.01
Constant	-3.01	2.20	0.92	1.29
Rho	-13.27***	0.91	-15.84*	9.14
N	198		253	

***p < 0.01, **p < 0.05, *p < 0.10

For the model based on the Roma sample, none of the three community-level variables were found to be statistically significant. With respect to the household- and individual-level factors, the only predictor of child marriage that emerged as statistically significant was age – with girls 15 years of age being significantly less likely to be currently married compared to girls 17 years of age, after controlling for other factors.

For both models, statistical tests of the endogeneity of current marriage and school enrollment status were conducted based on the correlation between the residuals for the two equations. If the correlation is zero (ρ = 0), this would indicate that the factors affecting the probability of being currently married and the factors affecting the probability of being enrolled in school are exogenous. However, if there is significant correlation between the residuals (ρ ≠ 0), then this indicates that common unobserved factors were influencing both probabilities, and that the two decisions were interdependent. For the nationally representative sample, ρ was statistically significant and negative (*p* = 0.0), as indicated in the second to last row in Table 2. For the model based on Roma girls, the statistical test on ρ was only marginally significant at the 10 % level (*p* = 0.09, outside the conventional threshold of *p* = 0.05).

## Discussion

The purpose of this study was to assess the risk factors associated with the practice of child marriage among females living in Roma settlements in Serbia, and to make comparisons with females living among the general population. The study contributes to the existing literature on child marriage in a number of ways. First, it is one of the few empirical studies of child marriage in a European setting. Second, it is one of the few studies that investigated child marriage among the Roma, and, by using data collected through a special purpose survey of households living in Roma settlements, avoided the problem of individuals not being willing to disclose their ethnic affiliation due to fear of discrimination. Finally, the study investigated whether child marriage and school enrollment decisions were interdependent, which to our knowledge has not yet been empirically explored in the child marriage literature.

The results indicated that, on average, Roma girls in Serbia were at very high risk of being married as children. Child marriage among Roma women in Roma settlements was also substantially higher than among women in the poorest wealth quintile in the general population. The vast majority of currently married Roma girls had partners who were less than 5 years older than themselves. However, for currently married girls in the overall population, the opposite is true – the vast majority had husbands more than five years older than themselves. The smaller spousal age gap among the Roma may be due in part to the fact that a greater percentage of Roma males are first married under age 18 than males in the general population [7].

Similar to the findings of a large number of previous studies, child marriage was found to be associated with a number of socio-economic characteristics, including household wealth, education, and urban/rural status [1, 3, 17]. While these findings suggest that economic factors may be playing an important role in influencing child marriage, it is important to emphasize that, for girls who married and moved away from their parent’s household to live with their husbands, we did not have information on the characteristics of the household where the girl grew up. It is possible that the relative socio-economic conditions of the individual’s present family and the family where she grew up may have been very different, particularly if girls tend to marry up the social ladder. We also explored whether child marriage was associated with community-level factors, as measured by the percentages of women in the community who: had secondary or higher levels of education; were first married under age 18; and who had zero tolerance of wife beating. These factors were found to be statistically significant among girls in the general population, but not among Roma girls, after controlling for other factors. The reason for the insignificance of these factors based on the Roma model estimations is unclear, but may be due to the low power of the analysis and the more limited variability in the community-level factors among this marginalized population. Urban/rural status was statistically insignificant after controlling for other factors in the model.

The tests for the endogeneity of current schooling and education based on the bivariate probit estimations provide some support for the hypothesis that decisions about girls’ current school attendance and child marriage are interdependent. For the nationally representative sample, that ρ is statistically significant (*p* = 0.0) suggests that common unobserved factors influence decisions about girls’ current school attendance and child marriage. For the sample of females living in Roma settlements, there was only weak evidence of the influence of common unobserved factors on both outcomes**,** as the statistical test on ρ is only marginally significant at the 10 % level (*p* = 0.09, outside the conventional threshold of *p* = 0.05). However, that rho is negative indicates that tradeoffs are being made between marriage and schooling in both groups, significantly more so among the general population, and that, in the Serbian context, it is not appropriate to disregard the possibility that school decisions are influenced by the timing of marriage. These findings are consistent with the arguments made by previous researchers regarding the interdependent nature of marriage timing and school enrollment decisions, due to the possibility that many of the same unobserved factors that influence the timing of marriage also influence schooling decisions [3, 14]. The factors could include parents’ educational attainment and aspirations for children, perceptions regarding the returns to schooling, availability of employment opportunities, and the degree of bargaining power of the girl [22].

The study has a number of limitations. First, the age and various outcomes investigated are self-reported and hence may be prone to bias due to social desirability and recall. Age of the respondent and age at marriage may be misreported. Second, for the bivariate probit models of the determinants of current marital status and current enrollment in school, the sample size of girls 15 to 17 years of age is relatively small, which reduced the power of the analysis. Third, the cross-sectional nature of the data prevented us from determining the actual temporal ordering of marriage and school enrollment decisions, nor did we have information on the types and characteristics of household members that played roles in the decisions. Fourth, the analysis of the determinants of child marriage and school enrollment includes a number of community-level indicators that were generated based on individual-level data from each sample cluster in the MICS data sets. While these factors may suggest the presence of a social norm, they do not necessarily establish the presence of a social norm. Furthermore, individuals’ perceptions of the boundaries of their community may not necessarily coincide with the boundaries of the sampling cluster. Individuals living in the same sampling cluster may not necessarily experience the same set of area influences especially in urban areas and outside the Roma settlements.

## Conclusions

The study finds evidence of the interdependence between marriage and school enrollment decisions among the general population and, to a lesser extent, among the Roma. Further research is needed on child marriage among the Roma and other marginalized sub-groups in Europe, and should be based on a mixed methods approach. Ideally, panel data that tracks girls over time should be used to assess the pathways leading to child marriage, including community-level factors and characteristics of the parents’ household. While panel data have some limitations, such as the loss of cases in the follow-up, such data are better suited for more rigorous analysis of the determinants and consequences of child marriage.

## Additional file

Additional file 1:
**Percentage of women 20 to 24 years of age living in the Roma settlements and general population samples who report first marrying under age 18, 2010.** (DOCX 80 kb)

## Abbreviations

MICS: multiple indicator cluster survey
UNICEF: United Nations Children’s Fund

## References

[1] UNICEF (2013). State of the World’s Children 2013: Children with Disabilities.

[2] Klugman J, Hanmer L, Twigg S, Hasan T, McCleary-Sills J, Santamaria J (2014). Voice and Agency: Empowering Woman and Girls for Shared Prosperity.

[3] Mensch BS, Singh S, Casterline JB, Lloyd CB, Behrman JR, Stromquist NP, Cohen B (2006). Trends in the timing of first marriage among men and women in the developing world. The changing transitions to adulthood in developing countries: Selected studies.

[4] Jain S, Kurz K (2007). New insights on preventing child marriage: A global analysis of factors and programs.

[5] Raj A, Boehmer U (2013). Girl child marriage and its association with national rates of HIV, maternal health and infant mortality across 97 countries. Violence Against Women.

[6] Open Society Foundations (2010). No Data—No Progress: Country Findings Data Collection in Countries Participating in the Decade of Roma Inclusion 2005–2015.

[7] Statistical Office of the Republic of Serbia (2011). Serbia Multiple Indicator Cluster Survey 2010.

[8] Idzerda L, Adams O, Patrick J, Schrecker T, Tugwell P (2011). Access to primary health care services for the Roma population in Serbia: a secondary data analysis. BMC Int Health Hum Rights.

[9] De Laat J (2010). Economic Costs of Roma Exclusion.

[10] Janevic T (2012). Socioeconomic position, gender, and inequalities in self-rated health between Roma and non-Roma in Serbia. Int J Public Health.

[11] Bosnjak B, Acton T (2013). Virginity and early marriage customs in relation to children’s rights among Chergashe Roma from Serbia and Bosnia. Int J Hum Rights.

[12] Pamporov A (2007). Sold like a donkey? Bride-price among the Bulgarian Roma. J R Anthropol Inst.

[13] Cvorovic J (2004). Sexual and reproductive strategies among Serbian Gypsies. Popul Environ.

[14] Bajracharya A, Amin S (2012). Poverty, marriage timing, and transitions to adulthood in Nepal. Stud Fam Plan.

[15] Das Gupta S, Mukherjee S, Singh S, Pande R, Basu S (2008). Knot ready: Lessons from programs and policies to delay marriage for girls in India.

[16] Lloyd CB, Mensch BS (2008). Marriage and childbirth as factors in dropping out from school: an analysis of DHS data from sub-Saharan Africa. Popul Stud.

[17] Mathur S, Greene M, Malhotra A (2003). Too young to wed: The lives, rights and health of young married girls.

[18] Speizer IS, Pearson E (2011). Association between early marriage and intimate partner violence in India: a focus on youth from Bihar and Rajasthan. J Interpers Violence.

[19] UNICEF (2005). Early marriage: a harmful traditional practice, a statistical exploration.

[20] Nour NM (2009). Child marriage: a silent health and human rights issue. Rev Obstet Gynecol.

[21] Becker G (1973). A theory of marriage. J Polit Econ.

[22] Lee-Rife S, Malhotra A, Warner A, Glinski AM (2012). What works to prevent child marriage: a review of the evidence. Stud Fam Plan.

