# Supplementary material for: Risk factors associated with the practice of child marriage among Roma girls in Serbia
Source: BMC Int Health Hum Rights. 2016 Feb 1;16:6. doi: 10.1186/s12914-016-0081-3 (PMC4736708; doi:10.1186/s12914-016-0081-3)
Supplement: Additional file 1: — Percentage of women 20 to 24 years of age living in the Roma settlements and general population samples who report first marrying under age 18, 2010. (DOCX 80 kb) [file 12914_2016_81_MOESM1_ESM.docx]

**Annex Table 1: Percentage of women 20 to 24 years of age living in the Roma settlements and general population samples who report first marrying under age 18, 2010.**

|  | **Roma** | | | **General Population** | | |
| --- | --- | --- | --- | --- | --- | --- |
| Variable | **%** | **95% CI** | | **%** | **95% CI** | |
| **Total** | 50.45 | [44.00, | 56.89] | 5.03 | [3.49, | 7.20] |
|  |  |  |  |  |  |  |
| **Residence** |  |  |  |  |  |  |
| Urban | 44.50 | [36.78, | 52.49] | 4.46 | [2.56, | 7.65] |
| Rural | 63.83 | [53.95, | 72.66] | 5.92 | [3.71, | 9.31] |
|  |  |  |  |  |  |  |
| **Regions** |  |  |  |  |  |  |
| Belgrade | 46.60 | [32.65, | 61.10] | 0.44 | [0.14, | 1.35] |
| Vojvodina | 50.84 | [38.21, | 63.36] | 7.29 | [3.94, | 13.08] |
| Sumadija and Western Serbia | 43.43 | [23.17, | 66.14] | 5.36 | [2.65, | 10.53] |
| Southern and Eastern Serbia | 54.27 | [45.98, | 62.33] | 8.02 | [4.32, | 14.39] |
|  |  |  |  |  |  |  |
| **Religion** |  |  |  |  |  |  |
| Orthodox Christians | 47.29 | [38.63, | 56.12] | 3.21 | [2.19, | 4.69] |
| Others | 54.51 | [45.67, | 63.07] | 22.46 | [12.07, | 37.95] |
|  |  |  |  |  |  |  |
| **Household wealth** |  |  |  |  |  |  |
| Poorest | 68.01 | [56.88, | 77.41] | 13.19 | [7.77, | 21.52] |
| Poorer | 56.44 | [44.29, | 67.85] | 8.05 | [4.03, | 15.44] |
| Middle class | 55.47 | [37.94, | 71.74] | 4.68 | [2.17, | 9.78] |
| Richer | 33.59 | [22.12, | 47.39] | 1.58 | [0.41, | 5.86] |
| Richest | 35.15 | [22.98, | 49.61] | 0.66 | [0.18, | 2.34] |
|  |  |  |  |  |  |  |
| **Women's education** |  |  |  |  |  |  |
| None | 59.55 | [46.19, | 71.64] | -- |  |  |
| Primary | 51.01 | [42.36, | 59.60] | 23.99 | [14.13, | 37.71] |
| Secondary | 30.5* | [17.41, | 47.75] | 5.72 | [3.60, | 8.96] |
| Higher | -- |  |  | 0.49 | [0.12, | 1.93] |
|  |  |  |  |  |  |  |
| N | 439 |  |  | 679 |  |  |
|  | | | | | | |
| *%: Weighted percentage* |  |  |  |  |  |  |
| *-- Indicates cell sizes <25* |  |  |  |  |  |  |
| ** Indicates cell sizes between 25-49* |  |  |  |  |  |  |
